# Supplementary material for: Large, long range tensile forces drive convergence during Xenopus blastopore closure and body axis elongation
Source: eLife. 2018 Mar 13;7:e26944. doi: 10.7554/eLife.26944 (PMC5896886; doi:10.7554/eLife.26944)
Supplement: Transparent reporting form [file elife-26944-transrepform.pdf]

eLIFE
1st Floor
24 Hills Road
Cambridge CB2 1JP, UK
P 01223 855340
W elifesciences.org
T @elife

eLife's transparent reporting form

We encourage authors to provide detailed information *within their submission* to facilitate the interpretation and replication of experiments. If you have any questions, please contact us: editorial@elifesciences.org.

Sample-size estimation

- You should state whether an appropriate sample size was computed when the study was being designed
- You should state the statistical method of sample size computation and any required assumptions
- If no explicit power analysis was used, you should describe how you decided what sample (replicate) size (number) to use

Please outline where this information can be found within the submission (e.g., page numbers or figure legends), or explain why this information doesn't apply to your submission:

When initially trouble-shooting tractor pull force measurements, conditions for running a tractor pull assay were modified until measurements were robust (relatively consistent, signal substantially greater than noise). Thereafter, a minimum of three error free runs (no explant failure, no tractor pull apparatus failure, no operator error) were measured. In many cases, more than 3 samples were used. This minimum was established to give some sense of statistical variation, while keeping in mind that setting up each tractor pull assay is a technically challenging, time-consuming procedure. We believe that the consistency of our results (see supplementary figure S4) indicates that this number is adequate for an initial assessment of the different force profiles for different types of explants.

For tractor pull stiffness measures, the same approach was taken, with an attempt to get a minimum of 3 runs for all types of measures; however under certain circumstances, certain types of explants at certain stages (late stage D180 and V180's) proved exceptionally difficult to get reliable measures from (difficulties detailed in the section in Supplementary Materials titled "**Caveats to stiffness measures of explants**" but we felt the measures we did obtain were of enough interest to be worth reporting.
In one instance we obtained one measure at an intermediate stage (for AC explants at 5.7 hours) that followed the overall trend but was otherwise uninteresting.

eLife Sciences Publications, Ltd is a limited liability non-profit non-stock corporation incorporated in the State of Delaware, USA, with company number 5030732, and is registered in the UK with company number FC030576 and branch number BR015634 at the address 1st Floor, 24 Hills Road, Cambridge CB2 1JP | August 2014

Replicates

- You should report how often each experiment was performed
- You should include a definition of biological versus technical replication
- The data obtained should be provided and sufficient information should be provided to indicate the number of independent biological and/or technical replicates
- If you encountered any outliers, you should describe how these were handled
- Criteria for exclusion/inclusion of data should be clearly stated
- High-throughput sequence data should be uploaded before submission, with a private link for reviewers provided (these are available from both GEO and ArrayExpress)

Please outline where this information can be found within the submission (e.g., page numbers or figure legends), or explain why this information doesn't apply to your submission:

Replicate numbers for Figure 3 are stated in the figure legend.

For Fig. 5A, individual data points are shown.
For Fig. 5B&C, the range of n's for each type of explant is reported in the figure legend; n's for each time point are as follows:
Standard Giants: n = 3 at 3 and 13 hours; n = 6 at 4.8 an 8.7 hours
Dorsal 180 explants: n = 4 at 3.4 hours, n = 2 at 7.8 and 11.9 hours
Ventral 180 explants: n = 5 at 4.3 hours, n = 3 at 7.6 hours, n = 2 at 12.3 hours
Animal Cap sandwich explants: n = 5 at 3.6 hours, n = 1 at 5.7 hours, n = 3 at 11.3 hours

For Tables 1 and 2, n's are reported in the table

For Tension Increase and Tension Relaxation experiments, and measures of elastic recoil reported in the Results sections titled "Tension developed by explants represents a progressively increasing, instantaneous stall force", n's are reported in the text for each experiment or measure.

For comparisons of stiffness measures between explants and times in the Results section titled "Structural stiffness increases in all tissues around the end of gastrulation", n's are reported in the text for each comparison.

The number of embryos assayed for the effectiveness of ventralization in the supplementary section titled "Immunohistochemistry & Effectiveness of Ventralization" is reported in the text.

Replicates, continued:

For Supplementary figure S1A:
Intact embryos: n = 1 at 0.5 and 9 hours; n = 3 at 1.5 hours; n = 5 at 2.4 and 5.4 hours; n = 6 at 3.4 hours; n = 7 at 4.5 hours; n = 2 at 7.3 hours.
Control Giants: n = 3 at 0 and 0.5 hours; n = 6 at 1.1 hours; n = 9 at 2 and 5 hours; n = 10 at 3 and 4 hours; n = 7 at 6 through 9 hours; n = 5 at 10.1 hours; n = 2 at 11.1 hours; n = 1 at 12.1 hours.
Std. Giant pull w/ probe #3: n = 3 at 0.9 hours; n = 5 at 1.9 and 6 through 16 hours; n = 6 at 3 and 4.9 hours; n = 7 at 3.9 hours.
Std. Giant pull w/ probe #4: n = 2 at 3.3, 21 and 22 hours; n = 4 at 4 through 18 hours; n = 3 at 19.1 and 20 hours.

For Supplementary figure 2B:
Equatorial circumference: n = 7 at 44 and 46 minutes; n = 4 at 45 and 50 through 111 minutes; n = 8 at 47 minutes; n = 3 at 127 through 304 minutes; n = 2 at 347 minutes
Subequatorial circumference: n = 5 at 44 and 47 minutes; n = 4 at 45, 46 and 50 through 111 minutes; n = 3 at 127 through 304 minutes; n = 2 at 347 minutes

For Supplementary figure S4:
Late Giant pulls mean, probe 3 & 4 combined: n = 4 at 5.1, 5.2 and 22.8 hours; n = 6 at 5.3 and 22.1 hours; n = 7 at 5.5 through 21.9 hours
UV Giant pulls mean, probe 3 & 4 combined: n = 2 at 3.6 through 3.9 hours; n = 3 at 4 through 4.3 hours; n = 5 at 4.4 through 4.7 hours; n = 6 at 4.8 through 5.9 and 20 through 22 hours; n = 7 at 6 through 7.6 and 9.6 through 19.9 hours; n = 8 at 7.7 through 9.5 hours
Ventral 180 pulls mean, probe 3 and 4 combined: n 4 at 3 through 3.3 and 17.9 through 22 hours; n = 5 at 3.4 through 4.0 hours and 14.4 through 17.8 hours; n = 6 at 4.1 through 14.3 hours

For Supplementary figure S9:
For all measures, n =3 at 3 and 13 hours and n = 6 at 4.8 and 8.7 hours

All replicates are biological replicates, which should be implicitly obvious, but is not explicitly stated.

Measures at successive time points for figure 3, were performed on the same explants, which should be implicitly obvious, but is not explicitly stated. Changes in the number of explants that means are reported for are indicated by breaks in the line.
Measures at successive time point for Fig. 5B&C were performed on the same explants, which is stated in the method section for “Structural Tensile Stiffness Measurement”.

Statistical reporting

- Statistical analysis methods should be described and justified
- Raw data should be presented in figures whenever informative to do so (typically when N per group is less than 10)
- For each experiment, you should identify the statistical tests used, exact values of N, definitions of center, methods of multiple test correction, and dispersion and precision measures (e.g., mean, median, SD, SEM, confidence intervals; and, for the major substantive results, a measure of effect size (e.g., Pearson's r, Cohen's d)
- Report exact p-values wherever possible alongside the summary statistics and 95% confidence intervals. These should be reported for all key questions and not only when the p-value is less than 0.05.

Please outline where this information can be found within the submission (e.g., page numbers or figure legends), or explain why this information doesn't apply to your submission:

For comparison of residual structural stiffness's in the section titled "Structural stiffness increases in all tissues around the end of gastrulation" (pg. 16, line 13), a paired t-test was used to compare the same giant sandwhich explants at 4.8 and 8.7 hours; (pg. 16, line 21) and an unpaired t-test was used to compare AC (ectodermal) sandwich explants at 3.6 and 11.4 hours (gastrula vs. neurula stages). N's are reported in the text.

For comparison of spring stiffness in the same section, (page 17, lines 12-13) a paired t-test was used to compare the same giant explant sandwiches at 4.8 vs. 8.7 hours (gastrula vs. midneurula) and an unpaired test was used to compare giant sandwiches at 8.7 vs. 13 hours (midneurula vs. closed late neurula). Unpaired t-tests were used to compare giant to AC sandwich explants (p17, line 16) at all stages and to compare giant to V180 sandwiches at all stage (p17, line 21). N's are reported in the text.

(For large datasets, or papers with a very large number of statistical tests, you may upload a single table file with tests, Ns, etc., with reference to page numbers in the manuscript.)

Additional data files ("source data")

- We encourage you to upload relevant additional data files, such as numerical data that are represented as a graph in a figure, or as a summary table
- Where provided, these should be in the most useful format, and they can be uploaded as "Source data" files linked to a main figure or table
- Include model definition files including the full list of parameters used
- Include code used for data analysis (e.g., R, MatLab)
- Avoid stating that data files are "available upon request"

eLife Sciences Publications, Ltd is a limited liability non-profit non-stock corporation incorporated in the State of Delaware, USA, with company number 5030732, and is registered in the UK with company number FC030576 and branch number BR015634 at the address 1st Floor, 24 Hills Road, Cambridge CB2 1JP | August 2014

Please indicate the figures or tables for which source data files have been provided:

My questions to editorial@elifesciences.org about how to format my extensive data files have gone unanswered, so I have not included anything in the current submission. My attempt to upload an excel file was unsuccessful, and if exported as a pdf file, some of the files run to 100 pages, which seems unwieldy. If someone could work with me to figure out how best to document and format this data, I would be happy to include the source files in a revision.
